# Disentangling Intrachain Folding from Interchain Assembly through Multidimensional Visualization

**DOI:** 10.1021/acs.jpcb.6c02030

**Published:** 2026-06-19

**Authors:** Murilo N. Sanches, Pritam Ganguly, Joan-Emma Shea, Vitor B. P. Leite

**Affiliations:** † Department of Physics, Institute of Biosciences, Humanities and Exact Sciences, 135132 São Paulo State University (UNESP), São José do Rio Preto, São Paulo 15054-000, Brazil; ‡ School of Chemistry and Materials Science, 8786 Rochester Institute of Technology, Rochester, New York 14623, United States; § Department of Chemistry and Biochemistry, University of California, Santa Barbara, California 93106, United States; ∥ Department of Physics, University of California, Santa Barbara, California 93106, United States; ⊥ Institute of Chemistry, 28108 São Paulo State University (Unesp), Araraquara, SP 14800-060, Brazil

## Abstract

Characterizing the conformational landscapes of intrinsically disordered proteins is essential for elucidating their roles in health and disease, but remains challenging due to their structural heterogeneity. In this study, we introduce an enhanced algorithmic framework for the Energy Landscape Visualization Method (ELViM) that enables the simultaneous mapping of monomeric and oligomeric conformational spaces within a unified metric space. Unlike traditional reaction-coordinate-based approaches, this extended ELViM implementation employs a distance-based similarity metric and force projection embedding to generate low-dimensional representations that preserve high-dimensional structural relationships without predefined bias. We expanded the standard workflow by integrating a density-guided Local Conformational Signature analysis coupled with a new consensus interchain contact mapping protocol. This development allows for the systematic disentanglement of intrachain folding dynamics from the interchain assembly interactions that drive protein aggregation. Using replica exchange molecular dynamics data for a 19-residue fragment of the tau protein, we demonstrate the method’s utility by comparing the conformational landscapes of the wild type and the aggregation-associated P301L mutant. ELViM effectively captures continuous conformational transitions and highlights key structural motifs, including the aggregation-prone PHF6 segment (VQIVYK). The analysis reveals how the P301L mutation acts as a structural stabilizer, shifting conformational preferences toward compact, preorganized ensembles with more persistent interchain contacts around PHF6. By providing a quantitative yet intuitive framework for exploring heterogeneous ensembles, this enhanced ELViM workflow offers broad applicability to studying folding landscapes, protein–protein interactions, and complex aggregation pathways.

## Graphical abstract

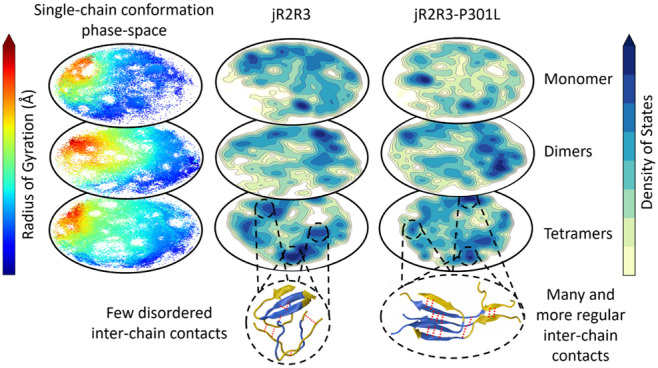

## Introduction

Understanding the conformational landscapes of biomolecules, particularly intrinsically disordered proteins (IDPs), is essential for elucidating their roles in health and disease. IDPs populate highly heterogeneous ensembles and lack a single dominant folded state, making it difficult to describe their behavior with traditional reaction-coordinate-based approaches.^1,2^ Such descriptions, while effective for systems with well-defined native structures, often fail to capture the breadth of structural diversity, hindering the identification of relevant conformational subpopulations, the detection of subtle transitions, and the connection of structural states to functional or aggregation pathways.^3–5^

The Energy Landscape Visualization Method (ELViM) provides a powerful framework for addressing these challenges. ELViM employs a metric based on internal residue–residue distances to quantify structural similarity, constructs a dissimilarity matrix, and projects it into a low-dimensional space using a force-based embedding scheme. This approach preserves structural relationships between conformations, enabling direct visualization of the conformational space without requiring predefined reaction coordinates. Coupled with density estimation and Local Conformational Signature (LCS) analysis, ELViM facilitates the identification of representative structures and structural motifs, providing an intuitive yet quantitative framework for exploring large-scale simulation data sets.^6,7^

To demonstrate the applicability of ELViM, we focus on a biologically and clinically relevant system: the microtubule-associated protein tau. Tau is an intrinsically disordered protein implicated in a range of neurodegenerative disorders collectively known as tauopathies, including Alzheimer’s disease and frontotemporal lobar degeneration.^8–10^ Human tau is a protein of over 400 amino acids that exists in isoforms containing either three or four imperfect repeat domains.^11^ In solution, tau remains disordered, but under pathological conditions, it undergoes misfolding and aggregation into insoluble amyloid fibrils, such as the paired helical filaments characteristic of Alzheimer’s disease.^12^ These fibrils are toxic due to their prion-like properties, enabling cell-to-cell propagation by templating the misfolding of soluble tau.^10,13^ However, the structural features that define a tau prion remain elusive, and the mechanisms by which monomeric tau transitions into fibrillar assemblies are not fully understood.

Given that tauopathy typically progresses over several decades in humans, experimental strategies designed to potentiate aggregation kinetics are essential for studying these mechanisms in the laboratory.^14^ Many of these models rely on disease-associated mutations, such as P301L, which are known to promote tau aggregation and prion-like propagation within experimentally tractable time scales.^15,16^ Despite its widespread use, the molecular-level effects of the P301L mutation on tau remain incompletely understood. A detailed comparison of the conformational landscapes sampled by the wild-type and P301L peptides, across both monomeric and oligomeric states, is therefore essential to identify the specific interactions modulated by this mutation.

In this study, we investigate a specific 19-residue tau fragment (residues from 295 to 313), given by DNIKHVP̲GGS**VQIVYK**PV, located at the junction of repeat number 2 and repeat number 3 domains in tau.^11,17^ This segment is crucial for aggregation as it contains the PHF6 hexapeptide (VQIVYK, bold above, residues 306–311), a critical element that forms the core of pathological tau fibrils.^18–21^ This fragment commonly adopts a strand–loop–strand conformation featuring two antiparallel β-strands in mature tau fibrils and is recognized as seed-competent, capable of inducing aggregation.^16,17,20^ It also harbors the previously mentioned disease-associated P301L mutation site, which promotes fibril formation and is linked to tauopathies such as frontotemporal dementia.^14,15,22^

This specific 19-residue tau fragment has been the subject of prior investigations, which established its key role in templated aggregation.^16^ Previous computational studies on this tau fragment, for instance, have explored how force field parameters influence the hydrophobic driving forces and water dynamics critical for peptide assembly, often by comparing its behavior across different force fields.^23^ Further experimental and computational work used this fragment to determine its aggregation competency, demonstrating that the P301L mutation enhances aggregation kinetics.^17^ However, these prior studies either characterized the ensemble using global metrics or focused on the propensity to aggregate. Our study shifts the focus to the structural consequences of the P301L mutation, aiming to elucidate the precise local conformational rearrangement and the specific fold adopted in the assembled state.

To elucidate the structural effects of the P301L mutation, we employed molecular dynamics simulations to generate conformational ensembles for both the wild-type peptide (hereafter referred to as jR2R3) and the P301L mutant (hereafter jR2R3-P301L) across monomeric, dimeric, and tetrameric states. We used capped termini (N-terminal acetyl and C-terminal *N*-methyl amide) for our initial molecular dynamics simulations. This modification is known to influence the conformational space sampled by IDPs differently than the uncapped versions.^18^ By using this approach, we provide complementary insights into the structural requirements necessary for stable fibril formation. The analysis was performed using the ELViM to map conformational landscapes, estimate population densities, and extract representative structures. Critically, the ELViM framework was extended to include interchain contact frequency maps for oligomers, enabling a systematic distinction between intra- and interchain contributions and a detailed assessment of how the P301L mutation reshapes aggregation relevant interactions.

## Methods

In the present work, the ELViM method was applied with a focus on the conformational phase space of each monomeric unit individually. This approach allows us to characterize the intrinsic structural variability of individual monomers in the context of their aggregation stage. The process of oligomerization and aggregate formation is addressed by explicitly considering intermonomeric contacts, thereby enabling a distinction between intrachain conformational dynamics and interchain interactions that drive assembly.

### ELViM: Energy Landscape Visualization Method

The Energy Landscape Visualization Method (ELViM) is designed to represent the conformational space of biomolecular systems without relying on a predefined reaction coordinate. The method consists of four main stages:

- Definition of a structural similarity metric to compare conformations.
- Construction of a dissimilarity matrix from the similarity scores.
- Dimensionality reduction using a force based projection scheme to generate a low dimensional visualization.
- Density of states and Local Conformational Signature (LCS) evaluation to identify representative structural motifs.

This workflow allows high dimensional simulation data to be reduced to an interpretable form while preserving structural relationships between conformations.

#### Structural Similarity Metric and Dissimilarity Matrix

To quantify the similarity between two conformations *k* and *l*, ELViM uses a metric based on the internal distances between all residue pairs:

1 $$q_{w}^{k,l}=\frac{1}{N_{\left(i,j\right)}}\underset{\left(i,j\right)}{\sum }\exp\left[\frac{-{\left(r_{i,j}^{k}-r_{i,j}^{l}\right)}^{2}}{2\sigma_{i,j}^{2}}\right]$$

where $r_{i,j}^{k(l)}$ is the distance between amino acids *i* and *j* of conformation *k*(*l*), *N* is the normalization factor ensuring *q* ^*k*,*l*^ is in the interval [0, 1], and σ_*i*,*j*_ = σ_0_|*i* – *j*|^ϵ^, where σ_0_ and ϵ are adjustable values. This equation was first presented by Wolynes and collaborators, where it was used for protein structure prediction.^24^ In ELViM, this measure is calculated for all pairs of structures *k* and *l* from a given trajectory, resulting in a symmetric *n* × *n*, where *n* is the total number of conformations analyzed.

The dissimilarity matrix is then computed as

2 $$\delta^{k,l}=1-q^{k,l}$$

where δ_*k*,*l*_ ranges from 0 (identical) to 1 (maximally different).

#### Multidimensional Projection

To visualize the energy landscape, the dissimilarity matrix is projected into two or three dimensions using the Force Scheme technique.^25^ In this method, points representing conformations are initially placed randomly and then iteratively displaced by attractive and repulsive forces until the projected distances match the original dissimilarities as closely as possible. Convergence is reached when positional changes fall below 10^–5^ between iterations.

The resulting projection preserves neighborhood relationships: nearby points correspond to structurally similar conformations, while distant points indicate structural dissimilarity.

#### Density Estimation and Local Conformational Signatures

The ELViM projection often contains regions of varying point density. To identify the most populated structural ensembles, we apply a Gaussian kernel density estimation, as implemented in SciPy.^26^ The kernel bandwidth can be adjusted to smooth the distribution and highlight significant regions.

From high density regions, we extract a Local Conformational Signature (LCS):

- Representative structures are chosen by computing the distance root-mean-square deviation (dRMSD) among conformations and selecting the centroid that minimizes this value.
- Neighboring structures are aligned to the centroid to form the LCS ensemble.
- Contact frequency maps are generated to identify recurrent intrachain or interchain interactions.

Unlike clustering methods, LCS analysis does not require partitioning the projection into discrete groups, allowing overlapping or gradient like transitions to be visualized.

#### Extension of the ELViM Approach

Up to this point, the analysis follows the standard ELViM framework as typically applied to single-chain proteins, focusing solely on intrachain conformational variability. However, in the case of oligomerization and aggregation, one must also account for interchain contacts and their influence on the conformational preferences of each monomeric unit. Addressing these interactions requires an additional set of procedures specifically designed to capture how inter monomer contacts reshape local conformational signatures and modulate the structural landscape of the assembly, specifically enabling the coupled analysis of intrachain structure and interchain interactions. The first procedural step is the independent monomer analysis within the ensemble, in which each chain is treated as an individual conformation. This step allows us to analyze the intrinsic structural variability and conformational contributions of every monomeric unit independently within the heterogeneous oligomeric ensemble. The second step is locating the chain pairs within the phase space, in which, after identifying regions of interest in the ELViM projection, an index is maintained to track which original trajectory conformations were in contact with the selected structures. The third step is a consensus contact map generation, in which contact frequency maps are generated for each pair of monomeric units (e.g., chain A against chain B, A against C, etc.) to quantify recurrent interchain interactions.

The third step is a consensus contact map generation, in which contact frequency maps are generated for each pair of monomeric units (e.g., chain A against chain B, A against C, etc.) to quantify recurrent interchain interactions, where a contact is defined by a distance of less than 8 Å between Cα atoms. Finally, the average frequency of each contact across all pairs is computed, yielding a single consensus map. This map characterizes how the conformations from a specific region of the ELViM phase space behave collectively in the context of oligomerization. This enhanced methodology allows for the distinction between intrachain conformational dynamics and the interchain interactions that drive assembly, providing a systematic way to assess how single-residue mutations reshape aggregation-relevant interactions across multiple assembly states. In this way, ELViM bypasses the reliance on arbitrary, predefined reaction coordinates, which can obscure subtle structural behaviors. Instead, the method per se generates a phase space distribution in which high-density regions accurately represent the most frequently sampled structures. This unbiased distribution can then be quantitatively probed to assess global structural characteristics. For instance, by associating each point’s position with its radius of gyration, ELViM enables a nonarbitrary identification of whether the resulting conformational distribution favors compact or extended monomeric units, providing a direct view of the sequence’s intrinsic structural tendencies.

### Application to Tau Protein Simulations

Replica-exchange molecular dynamics (REMD) simulations were performed for the jR2R3 and jR2R3-P301L peptides.^27–29^ The temperature ladder spanned 292.7–455.6 K, and 64 replicas were used in each simulation. Exchange probabilities between neighboring replicas remained within 0.2–0.3. All calculations were conducted with GROMACS 2021.2.^30^ Each system was placed in a dodecahedral simulation box with an approximate linear dimension of 8 nm. About 12,000 water molecules were included for the monomeric and oligomeric systems. One, two, or four peptide chains were simulated for the monomer, dimer, and tetramer cases, respectively. To maintain overall charge neutrality, one chloride ion was added per peptide. Monomer and dimer REMD trajectories were run for 500 ns, and tetramer simulations were run for 800 ns. Only the final 300 ns of the REMD trajectories were used for data analysis.

Initial peptide structures were extracted from the cryo-EM tau fibril model associated with CBD pathology.^31^ The peptide termini were capped with an acetyl group at the N-terminus and an *N*-methyl amide at the C-terminus. The CHARMM36m force field was used for the peptides, in conjunction with the CHARMM-modified TIP3P water model.^32–34^ Before initiating REMD, each system underwent 5 ns of NPT equilibration at 300 K and 1 bar using the Berendsen thermostat (0.5 ps relaxation) and the Berendsen barostat (3 ps relaxation).^35^ Temperature control during the production REMD (NVT ensemble) was achieved using the Nosé–Hoover thermostat with a 3 ps relaxation time.^36,37^ A 1.2 nm cutoff was used for nonbonded interactions. Long-range dispersion corrections were applied to both energy and pressure. Long-range electrostatics were handled using Particle-Mesh Ewald with a grid spacing of 0.12 nm.^38^ Bond constraints for peptides were maintained using the LINCS algorithm, and water geometry was preserved using SETTLE.^39,40^ A 2 fs time step and the leapfrog integrator were employed.^41^

For each aggregation state, 15000 conformations from jR2R3 and 15000 from jR2R3-P301L were equally spaced and selected for analysis. In the case of dimers and tetramers, only structures containing at least one interchain hydrogen bond were included to exclude dissociated states. ELViM was then used to project the conformational space, estimate densities, and extract LCSs, enabling direct visual comparison between jR2R3 and mutant structural landscapes.

## Results and Discussions

Figure 1 presents the effective phase spaces for each simulated degree of aggregation. The circular symmetry is a property that emerges from the force scheme algorithm and the data used. In this figure, the points are colored according to the radius of gyration values of each represented structure, showing that these conformational spaces vary smoothly across the projections, indicating that distances between conformations are consistent with the Force-Scheme minimization goals.^7^ There are, however, due to the nature of the data used, instances in which there is no circular symmetry.^42^ The variation of the radius of gyration demonstrates that, in all three systems, there is a distribution of regions with extended structures (upper left) diametrically opposed to regions with collapsed structures (lower right). It is important to note that the X and Y axes in the ELViM projection do not correspond to any physical quantity, but rather provide abstract embedding dimensions where proximity reflects structural similarity.

**Figure 1:**
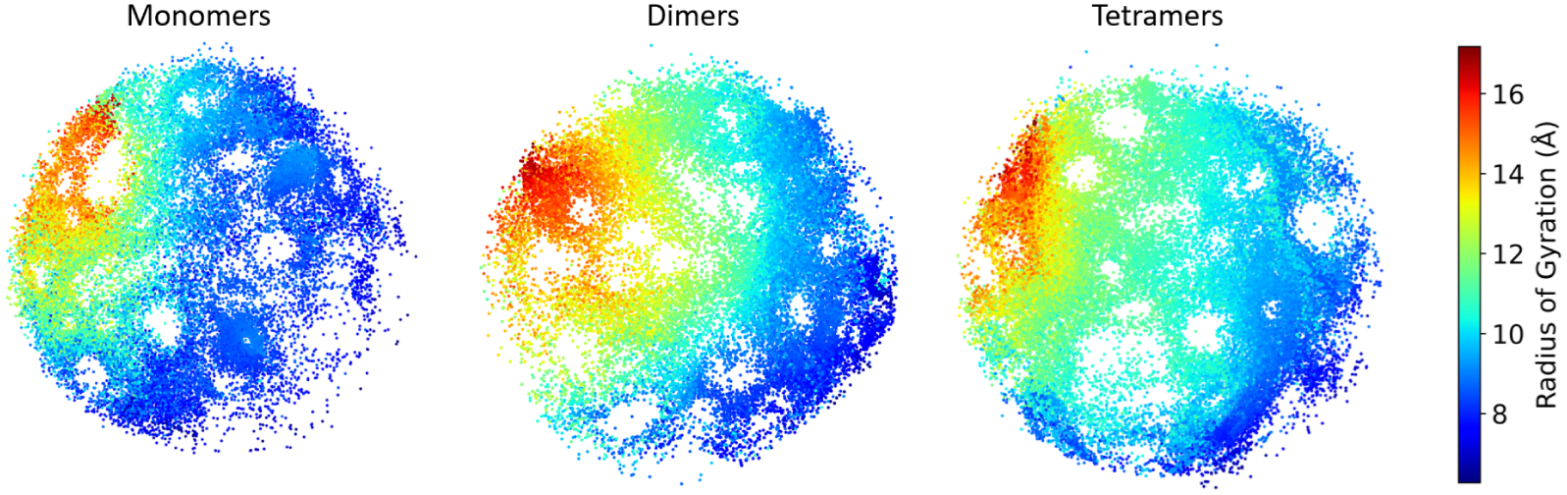
Effective phase space of each aggregation state projected by ELViM. The projections contain the structural ensembles sampled for both jR2R3 and jR2R3-P301L in aqueous solution. The points are color-coded according to the radius of gyration.

To distinguish the states accessed by each system (jR2R3 or jR2R3-P301L), we used LCS analysis. First, we estimated the density of points in the projection. Figures 2A and 2C show the density of monomers for the jR2R3 and jR2R3-P301L points, respectively, with a color scale ranging from white, indicating zero density, to dark blue, indicating regions of high density. Next, we selected the densest regions and extracted their 50 most representative structures, which are shown superimposed around their respective projections. Based on the selected conformations, we generated intrachain contact frequency maps for each selected region, displayed in Figures 2B and 2D for jR2R3 and jR2R3-P301L, respectively. These maps indicate how frequently intrachain contacts were observed, with 100% (dark blue) meaning the contact was present in all 50 structures analyzed for that region. The upper half of each map corresponds to Region I, and the lower half to Region II (highlighted in Figures 2A and 2B).

**Figure 2:**
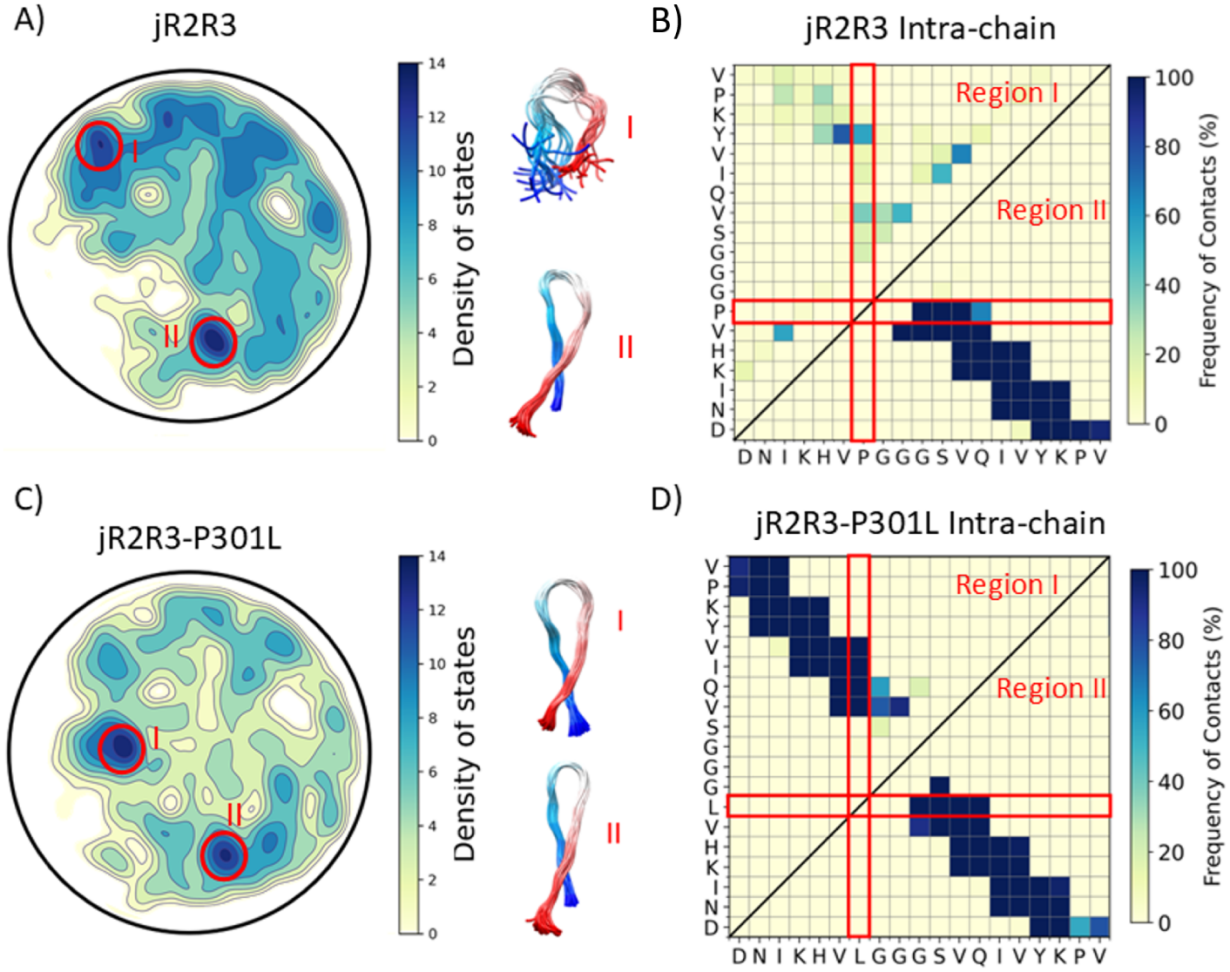
LCS analysis for the monomers of the Tau fragment. A) ELViM projection with point density estimation for the jR2R3 structures, with the two densest regions highlighted in red and the corresponding structures shown around the projection. B) Intrachain contact frequency map of the conformations selected in A), with the upper half corresponding to Region I and the lower half to Region II. C) ELViM projection with point density estimation for the jR2R3-P301L structures, with the two densest regions highlighted in red and the corresponding structures shown around the projection. D) Intrachain contact frequency map of the conformations selected in C), with the upper half corresponding to Region I and the lower half to Region II.

These analyzes reveal which structures were most frequently sampled throughout the simulations, as well as the role of residue P301 in stabilizing these conformations. In the case of jR2R3 (Figure 2A), we observed that structural ensemble II was sampled more frequently. This conformation is characterized by a β-prone extended topology. Upon analyzing the contact frequency in Region II, we see that all but one contact occurred with 100% frequency, indicating that these interactions were consistently maintained across the structures. Particularly, three of these contacts involved residue P301. This contrasts with the analysis of Region I, the second most densely populated area for jR2R3, which did not exhibit full alignment by dRMSD, as illustrated by structural ensemble I. This behavior is reflected in the contact frequency of Region I, which shows only six contacts with frequencies above 60%, and only one of them involves residue P301, indicating greater disorder. In contrast, the density analysis for the jR2R3-P301L shows that the two most frequently sampled ensembles exhibited good structural alignment, with a slight “shift” between them. This becomes more apparent when comparing the contact maps, which reveal a two residue offset between the contact signatures of Regions I and II. The contact maps also reveal a distribution of contacts perpendicular to the diagonal, reflecting a back-folded topology within the monomer. Furthermore, in both ensembles, the contact frequencies remain consistently above 95%, with residue P301 stabilizing four of these specific interactions.

These results show that jR2R3 explores a broader and more heterogeneous conformational space, whereas jR2R3-P301L is confined to more compact ensembles with stable contact patterns of a back-folded topology. ELViM thus provides a clear view of how residue P301 modulates monomeric conformations, setting the stage for analyzing how interchain interactions reshape their structural ensembles, as shown in Figure 3, for the dimers conformations. Again, after estimating the point density of the projection, we selected the 50 most representative structures from the two densest regions of each system. The analysis reveals that the individual conformations of each jR2R3 chain (Figure 3A) resemble structures found in mature fibrils, with the contact map of Region I in Figure 3B exhibiting contacts (with frequencies above 80%) usually found on amyloid conformations. In contrast, for Region II, these contacts are not fully evident (with lower frequencies) due to structural variance, with the average contact frequency hovering around 60%. We then performed the additional analysis to investigate interchain contacts, shown in Figure 3C and D for jR2R3 Regions I and II, respectively. In Region I, jR2R3 conformations exhibit frequent contacts between chains A and B, confirming aggregation, while Region II shows weaker interactions, particularly between the residues G304 to V309, which are not consistently preserved across conformations. For the jR2R3-P301L (Figure 3E), the representative structures did not resemble fibrils as in jR2R3: Region I retains a β-strand, whereas Region II displays fully extended conformations with minimal intrachain contacts (Figure 3F). Nonetheless, inspection of interchain contacts reveals strong terminal interactions in Region I, present in all 50 analyzed structures (Figure 3G), and in Region II both chains remain in contact along their length (Figure 3H). In both regions, the mutated residue forms frequent contacts (90–100%), potentially stabilizing interchain interactions. Notably, interchain contacts in the extended jR2R3-P301L conformations, as well as in jR2R3, are located around the aggregation prone PHF6 motif, a key element forming the core of pathological tau fibrils,^18,20^ whereas the β-strands jR2R3-P301L structures lack contacts in this region. The βs-strands conformations of jR2R3-P301L may prevent interchain contacts around the PHF6 motif due to their compact geometry, which shields the motif and limits its exposure to neighboring monomers. This structural arrangement could hinder the alignment necessary for aggregation, reducing the likelihood of forming the contacts that are typically observed in jR2R3 or extended jR2R3-P301L conformations.

**Figure 3:**
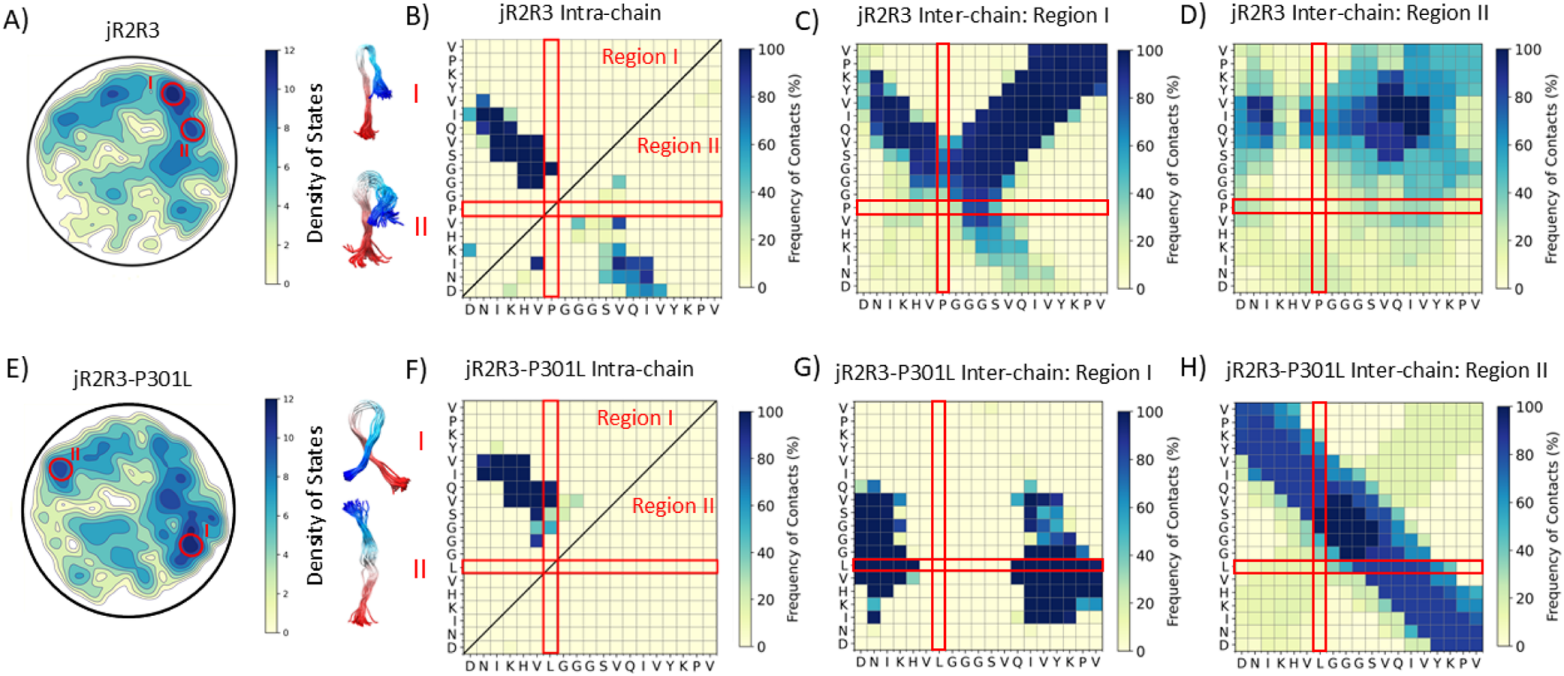
LCS analysis for Tau fragment dimers. A) ELViM projection with point density estimation of jR2R3 structures, highlighting the two most populated regions in red, with their corresponding structures shown around the projection. B) Intrachain contact frequency map of the conformations selected in A), with the upper half corresponding to Region I and the lower half to Region II. C) Interchain contact frequency map for the structures selected in A), Region I, showing contacts with the opposite chain in the simulation. Residue indices on the X-axis correspond to chain A, and those on the Y-axis correspond to chain B. D) Interchain contact frequency map for the structures selected in A), Region II, with the same axis assignments as in C). E) ELViM projection with point density estimation of jR2R3-P301L structures, highlighting the two most populated regions in red, with their corresponding structures shown around the projection. F) Intrachain contact frequency map of the conformations selected in E), with the upper half corresponding to Region I and the lower half to Region II. G) Interchain contact frequency map for the structures selected in E), Region I, showing contacts with the opposite chain in the simulation. Residue indices on the X-axis correspond to chain A, and those on the Y-axis correspond to chain B. H) Interchain contact frequency map for the structures selected in E), Region II, with the same axis assignments as in G).

The dimer analysis elucidates how interchain contacts shape the structural ensembles: jR2R3 preferentially forms contacts around the aggregation-prone PHF6 motif, whereas jR2R3–P301L exhibits conformation-dependent interaction patterns. ELViM effectively resolves these differences in phase space, capturing both density distributions and local conformational signatures, and provides a robust framework for extending the analysis to higher-order assemblies, such as tetramers. To complement this analysis, the most representative full-complex structures for both regions of the jR2R3 and jR2R3–P301L dimers are presented in the Supporting Information (SI) as Figure S1, which highlights the relative orientations of chains A and B.

Figure 4 presents the application of the previously described ELViM based protocol to tetrameric chains. Unlike the dimers, jR2R3 conformations are almost entirely extended, exhibiting only a few localized intrachain contacts (Figure 4B). For intrachain contact analysis, we calculated the average interactions among each chain (A, B, C, and D) as well as the average across both high density regions, thereby minimizing the influence of structural variations on the aggregation assessment. This approach yields a single contact frequency map (Figure 4C), which shows that although contacts are observed among all four chains, their maintenance frequency rarely exceeds 60% of the analyzed conformations. In contrast, the mutated structures reveal two distinct ensembles (Figure 4D). The individual interchain map between each chain is available in the SI as Figures S2, S3, S4 and S5 for the jR2R3 Region I, jR2R3 Region II, jR2R3-P301L Region I and jR2R3-P301L Region II respectively. From this analysis, Region I retains the conformations with a characteristic β-strand contact pattern (Figure 4E), whereas Region II displays highly extended structures with no intrachain contacts. Interchain interactions in jR2R3-P301L are stronger than in jR2R3, with contacts present in 100% of the analyzed conformations, and the average contact map highlights the highest frequency of interactions around the aggregation prone PHF6 motif. In contrast, jR2R3 tetramers show very few preserved interchain contacts between conformations, indicating weaker and less consistent oligomerization. Notably, no specific involvement of the mutated residue was observed in stabilizing these interactions. Visualizations of the full tetrameric assemblies, illustrating the collective arrangement of all four monomeric units for the primary density regions of each system, are presented in Figure S6 of the SI.

**Figure 4:**
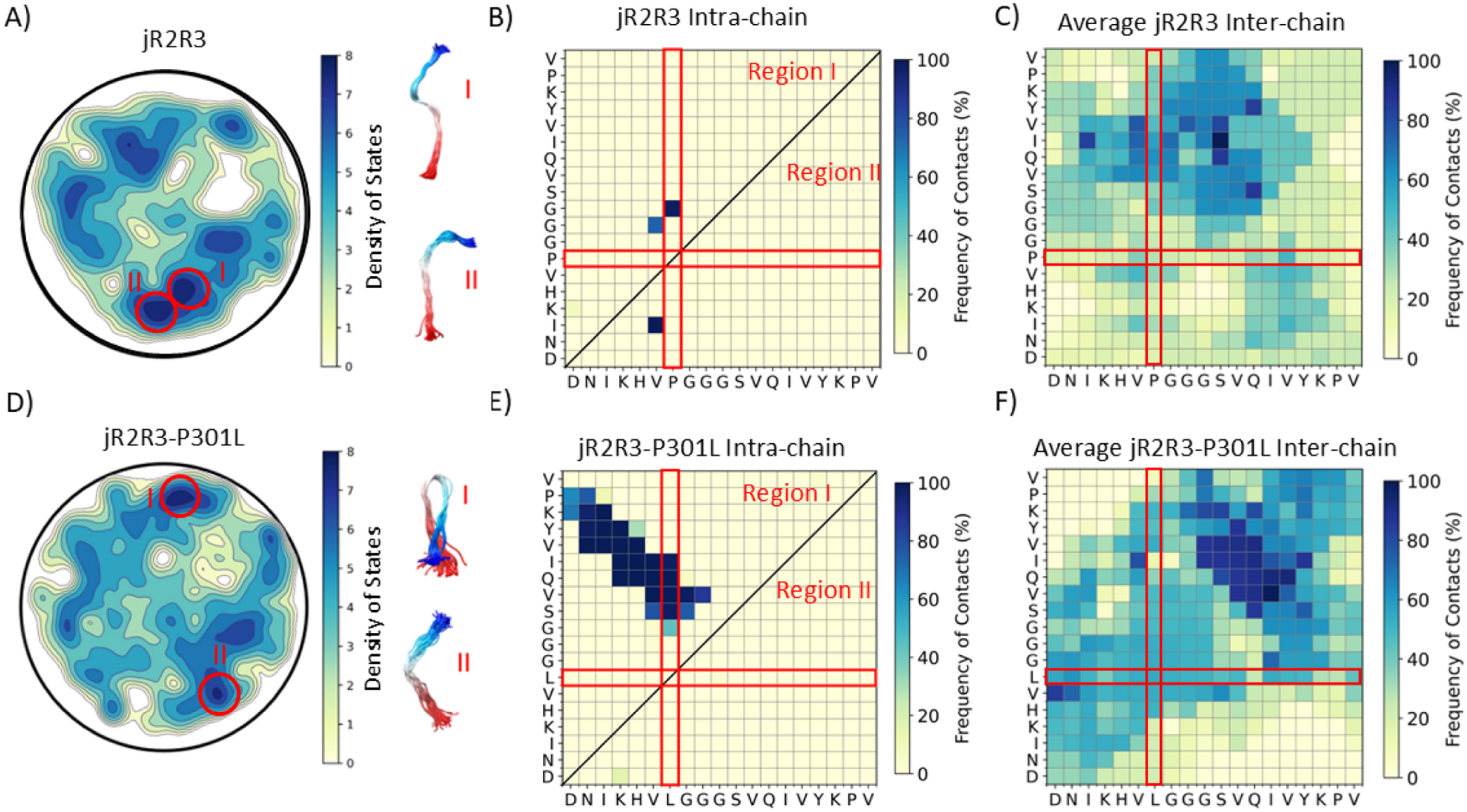
LCS analysis for the Tau fragment tetramers. A) ELViM projection with the estimated point density of the jR2R3 structures, highlighting the two densest regions in red along with their corresponding structures around them. B) Intrachain contact frequency map of the conformations selected in A), with the upper half corresponding to Region I and the lower half to Region II. C) Average interchain contact frequency map of the structures selected in A), showing interactions with the other chains in the tetrameric complex. D) ELViM projection with the estimated point density of the jR2R3-P301L structures, highlighting the two densest regions in red along with their corresponding structures around them. E) Intrachain contact frequency map of the conformations selected in D), with the upper half corresponding to Region I and the lower half to Region II. F) Average interchain contact frequency map of the structures selected in D), showing interactions with the other chains in the tetrameric complex.

### Mechanism of Early-Stage Aggregation

The mechanism of early-stage aggregation can be revealed by ELViM’s analysis of contacts across aggregation states. This analysis provides a distinct, stepwise picture of how the P301L mutation drives Tau aggregation by facilitating this pathway. In the monomeric state, the jR2R3 peptide samples a broad, heterogeneous conformational space with less consistent intrachain contacts, indicating greater disorder. In contrast, the jR2R3-P301L mutant is restricted to more compact ensembles with contacts that reflect a back-folded topology. The P301L residue acts as a structural stabilizer by highly involving itself in and stabilizing four cross-strand intrachain contacts with residues in the PHF6 motif (specifically, L301 with V306, Q307, I308 and V309), which reflects the importance of specific sequence patterns in amyloid formation.^43^

We note that in this work, we employed capped termini (acetyl at the N-terminus and *N*-methyl amide at the C-terminus), a modification known to influence the conformational space sampled in intrinsically disordered proteins compared to uncapped versions.^18^ While uncapped simulations often result in β-hairpins due to terminal electrostatic attractions, our capped simulations allow for increased distances between the terminals, enabling the peptide to sample arc-like (u-shaped) and more extended structures. Hence, we identified more extended and u-shaped populations of jR2R3 and jR2R3-P301L monomers and dimers here than in our earlier work with capped peptides in which we observed a greater propensity to form β-hairpin topologies, particularly in the case of the wild-type jR2R3 variant.^16,17,23^

Across all subsequent oligomeric state (dimer and tetramer), the analysis reveal a stark contrast: jR2R3 structures remain largely extended with weak and inconsistent interchain contacts. However, jR2R3-P301L ensembles exhibit extended conformations where interchain interactions are highly frequent and concentrated specifically around the aggregation-prone PHF6 motif. Notably, in the dimeric state, we identified populations of extended jR2R3-P301L dimers. By successfully disentangling the effects of jR2R3-P301L on both intra- and interchain interactions and by offering visualization of the continuous conformational landscape, ELViM provides crucial mechanistic insight into these early aggregation events. This approach demonstrates the structural basis for enhanced aggregation upon mutation, where the P301L mutation favors a β-strands conformation that is ideally preorganized for early-stage aggregation via stable PHF6 contacts.

## Conclusion

This work establishes ELViM as a versatile and comprehensive framework for exploring, visualizing, and interpreting the conformational landscapes of complex biomolecular systems, allowing detailed structural analysis of each state along the aggregation pathway. By combining a distance-based similarity metric with multidimensional projection and density-guided LCS analysis, ELViM enables an intuitive representation of structural ensembles while preserving essential information about local and global conformational relationships, capturing both monomeric variability and interchain contacts in oligomers. Unlike traditional methods that rely on predefined reaction coordinates or discrete clustering, ELViM captures the continuous and overlapping nature of conformational space, allowing subtle transitions, rare states, and gradient-like features to be identified and quantified.

The application of the enhanced ELViM framework has provided crucial mechanistic insights into the early stages of tau aggregation, successfully disentangling the effects of the P301L mutations across assembly states. As opposed to standard methods, the ability to couple intrachain structure with interchain contact analysis revealed that the P301L mutation confers a mechanistic advantage by preorganizing the monomer into a β-strand conformation. The analysis highlighted clear differences between the jR2R3 and the jR2R3-P301L: while jR2R3 structures maintain weak and inconsistent interchain contacts, extended jR2R3-P301L conformations strongly favor persistent and high frequency interchain contacts concentrated specifically around the PHF6 motif. This finding confirms that the jR2R3-P301L induced monomeric structure serves as an ideal structural template for early-stage aggregation via stable PHF6 contacts.

It is worth noting that while this 19-residue segment acts as an intrinsic structural module driven by local sequence propensities, this context operates within a broader global framework. Significantly shortening the fragment would eliminate critical flanking residues (such as position 301) necessary to maintain its native topology. Conversely, in full-length tau, larger surrounding disordered regions do not alter these intrinsic propensities, but rather form transient long-range contacts or modulate accessibility during transition into interchain assemblies.

Overall, these results underscore the critical importance of local conformational context and sequence variation in modulating aggregation propensity, establishing the structural basis for enhanced aggregation and guiding future therapeutic efforts.

Additionally, while ELViM operates primarily as a nonparametric postprocessing framework without a continuous analytical gradient for direct simulation biasing, its structural landscapes serve as a robust benchmark for validating sampling convergence. By isolating discrete conformational basins without predefined geometric metrics, it offers a rigorous method for selecting discrete structural seeds to optimize ensemble-based sampling protocols in highly heterogeneous biopolymers.

Beyond tau, ELViM’s conceptual and computational framework is broadly applicable to a wide array of biomolecular systems. Its integration of structural similarity, projection, and density-guided analysis makes it particularly suited for studying intrinsically disordered proteins and aggregation pathways, providing a bridge between raw simulation data and mechanistic understanding.

## Supplementary Material



## Data Availability

Comprehensive details regarding the molecular dynamics simulations and the specific analysis protocols (including the extended ELViM framework and consensus contact mapping) are documented in the Methods section. To support transparency and reproducibility, the REMD trajectories, Python-based analysis scripts, and raw data for both the jR2R3 and jR2R3-P301L systems are openly available on Zenodo (DOI: 10.5281/zenodo.19317264).

## Notes

Figure S1: representative full-complex structures for the dimeric assemblies of jR2R3 and jR2R3-P301L, showing the relative orientations of both chains A and B in Regions I and II; Figures S2 and S3: individual interchain residue–residue contact maps for jR2R3 (Regions I and II, respectively); Figures S4 and S5: individual interchain residue–residue contact maps for the jR2R3-P301L mutant (Regions I and II, respectively); Figure S6: representative full-complex structures for the tetrameric assemblies of jR2R3 and jR2R3-P301L, illustrating the collective arrangement of all four monomeric units for the primary density regions; Figures S2–S5: individual interchain contact maps for the jR2R3 and jR2R3-P301L constructs to supplement the consensus maps shown in the main text (PDF)
