# Supplementary material for: Disentangling Intrachain Folding from Interchain Assembly through Multidimensional Visualization
Source: J Phys Chem B. 2026 Jun 19;130(26):6577–85. doi: 10.1021/acs.jpcb.6c02030 (PMC13339632; doi:10.1021/acs.jpcb.6c02030)
Supplement: Supplementary file 1 [file jp6c02030_si_001.pdf]

Supporting Information - Disentangling Intra-chain Folding from Inter-chain Assembly through Multidimensional Visualization

Murilo N. Sanches,[†,†] Pritam Ganguly,[‡] Joan-Emma Shea,[*,¶,§] and Vitor B.P. Leite[*,‖]

†*Department of Physics, São Paulo State University (UNESP), Institute of Biosciences, Humanities and Exact Sciences, São José do Rio Preto, SP, 15054-000, Brazil*

‡*School of Chemistry and Materials Science, Rochester Institute of Technology, Rochester, New York 14623, United States*

¶*Department of Chemistry and Biochemistry, University of California, Santa Barbara, California 93106, United States*

§ *Department of Physics, University of California, Santa Barbara, California 93106, United States*

‖*Institute of Chemistry, São Paulo State University (UNESP), Araraquara, SP, 14800-060, Brazil*

E-mail: shea@ucsb.edu; vitor.leite@unesp.br

Supporting Figures

This section provides a characterization of the tau fragment assemblies. We first present the representative (defined by dRMSD) full-complex conformations for the jR2R3 and jR2R3-P301L systems in the dimeric states (Figure S1). These snapshots illustrate the relative spatial orientation of the chains, with the aggregation-prone PHF6 motif (residues 306–311) highlighted in blue to facilitate comparison across different conformational regions.

Next, we present the inter-chain contact maps between all the chains for the tetramers conformations. We selected 50 structures from the regions highlighted in Figures 4 of the main text. Then, we generated a contact frequency map for each pair of monomeric units (chain A against chain B, B against C, etc.), defining a contact by a distance of less than 8 Å between $C\alpha$ atoms. High-frequency contacts (indicated in dark blue) represent stable interactions, while lighter regions indicate more transient or distant residue pairings.

Lastly, Figure S6 illustrates the representative full-complex conformations in the tetrameric states.

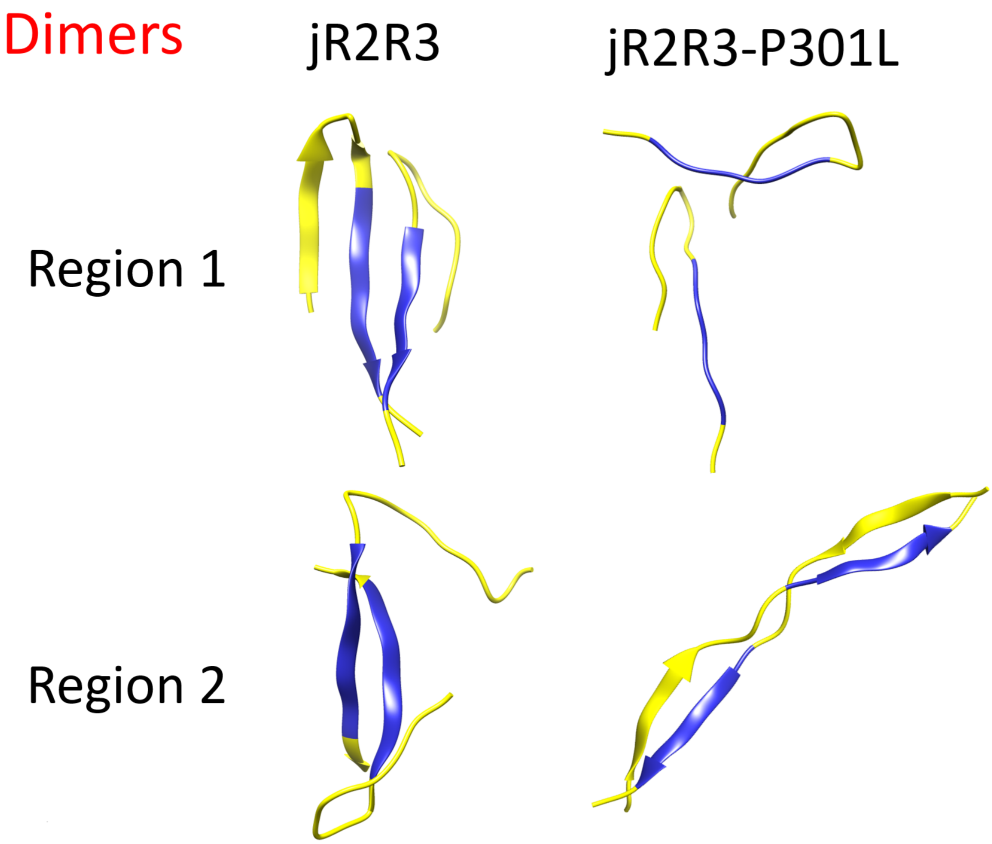

Figure S1: Representative full-complex conformations are shown for the jR2R3 and jR2R3-P301L dimers extracted from the two most populated regions of the ELViM phase space. The aggregation-prone PHF6 motif (residues 306–311) is highlighted in blue to illustrate its orientation and involvement in inter-chain assembly.

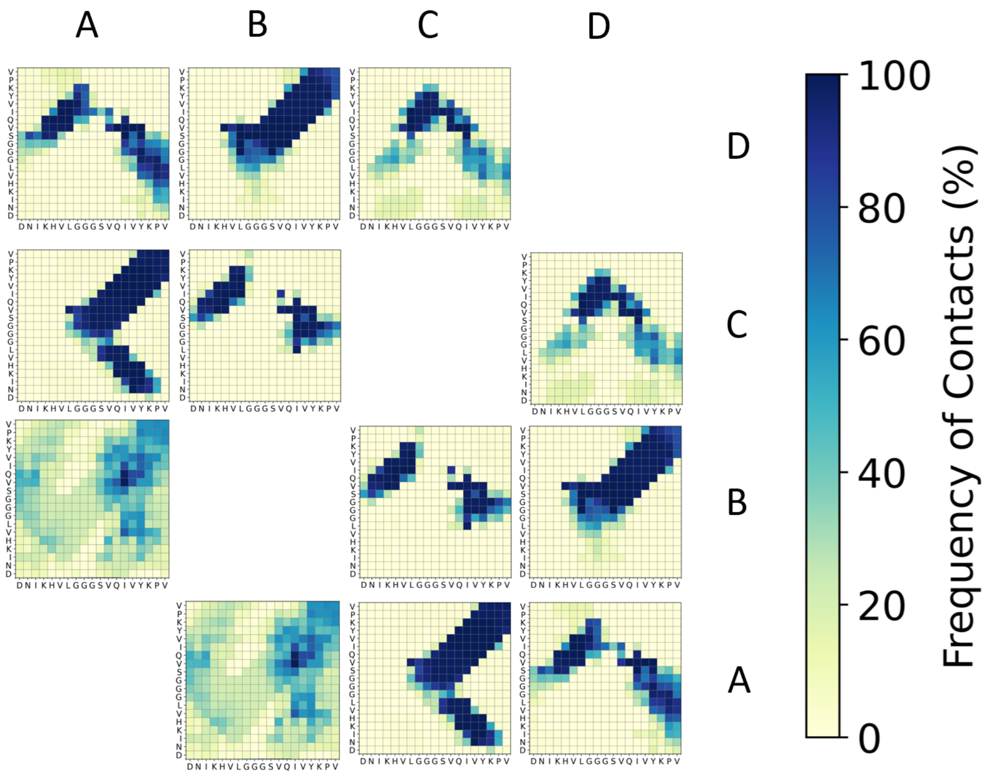

Figure S2: Inter-chain contact frequency maps for jR2R3. The plots show the frequency of contacts in percent between residues in one jR2R3 chain (y-axis) and residues in the neighboring jR2R3 chain (x-axis) for the structures selected in Region I from Figure 4 of the main text.

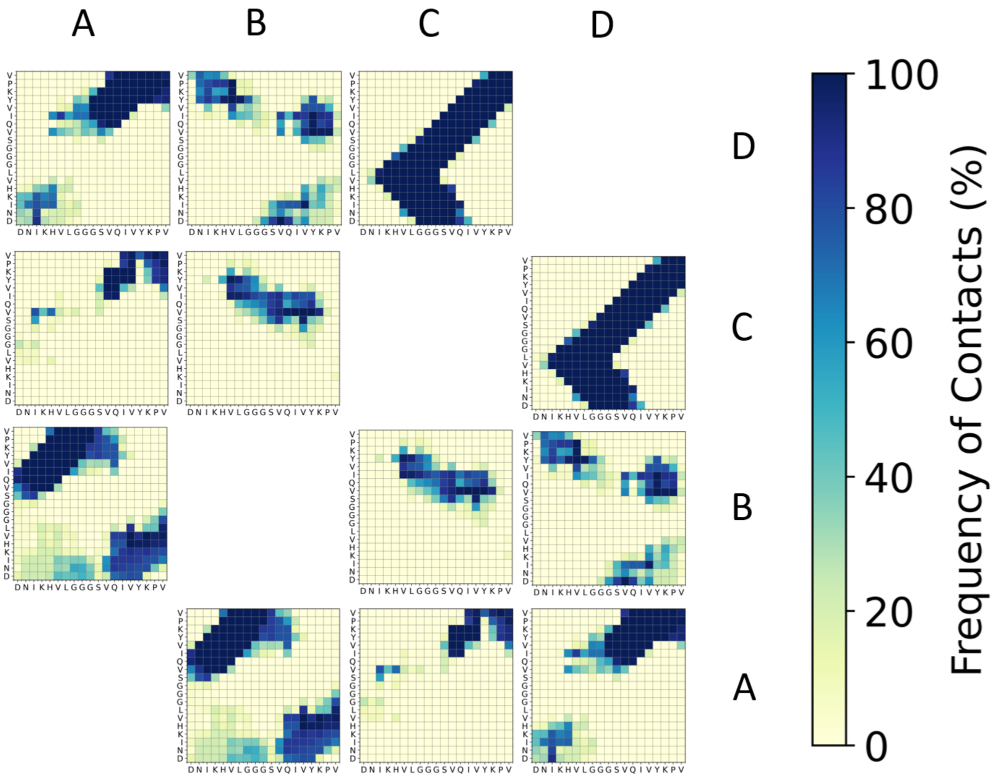

Figure S3: Inter-chain contact frequency maps for jR2R3. The plots show the frequency of contacts in percent between residues in one jR2R3 chain (y-axis) and residues in the neighboring jR2R3 chain (x-axis) for the structures selected in Region II from Figure 4 of the main text.

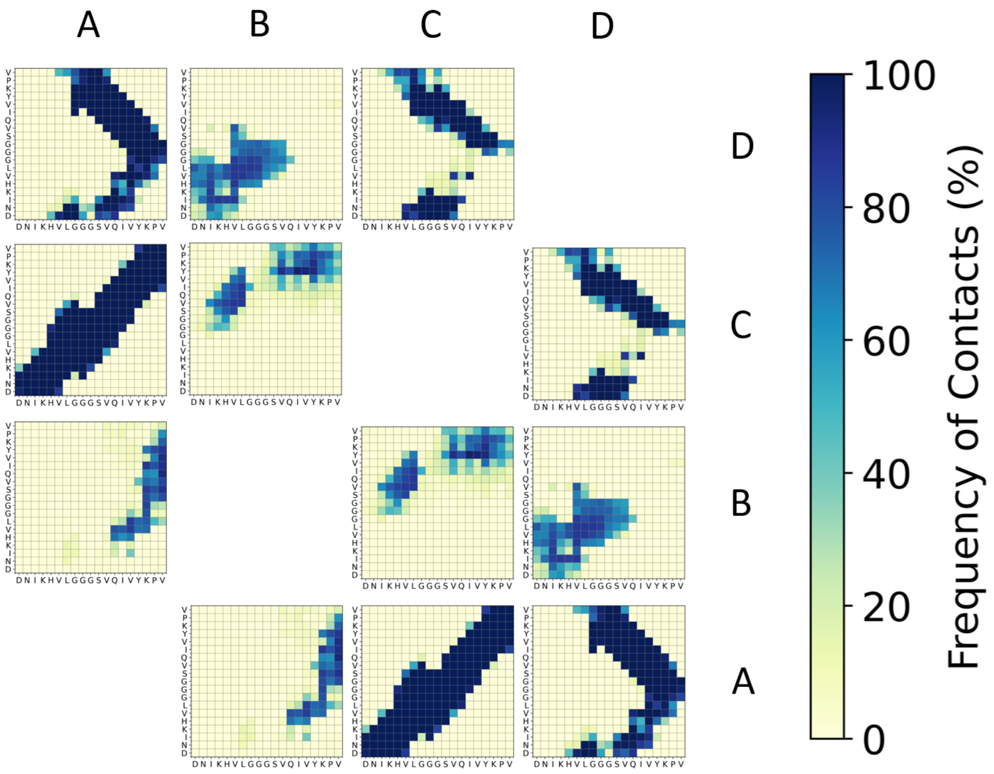

Figure S4: Inter-chain contact frequency maps for jR2R3-P301L. The plots show the frequency of contacts in percent between residues in one jR2R3-P301L chain (y-axis) and residues in the neighboring jR2R3-P301L chain (x-axis) for the structures selected in Region I from Figure 4 of the main text.

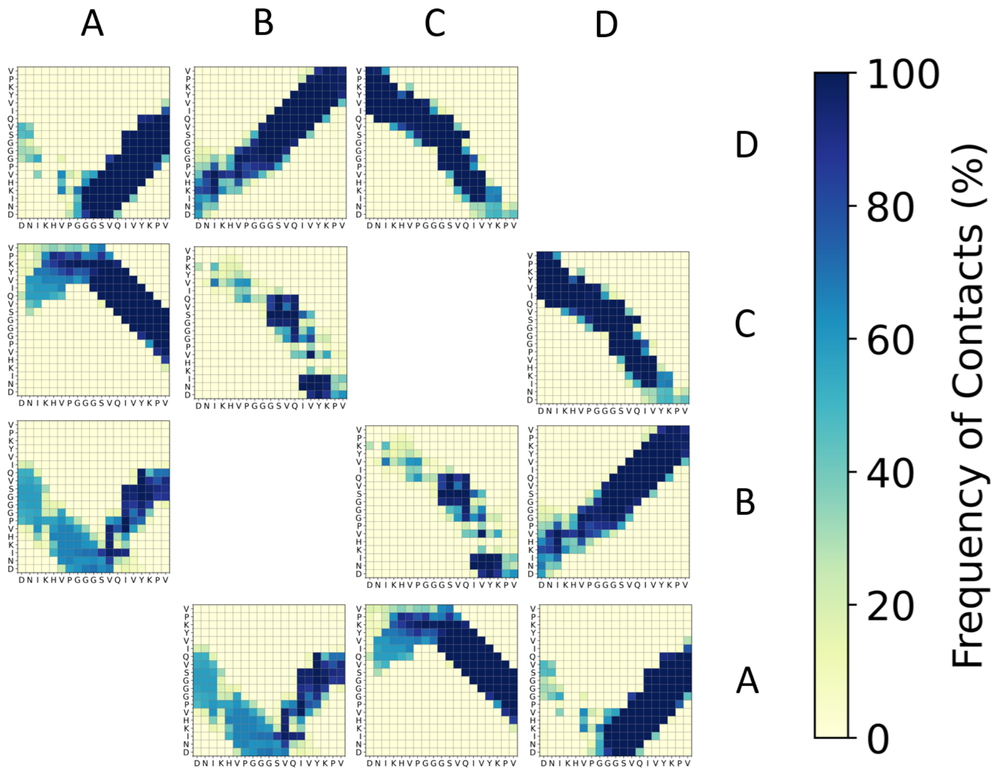

Figure S5: Inter-chain contact frequency maps for jR2R3-P301L. The plots show the frequency of contacts in percent between residues in one jR2R3-P301L chain (y-axis) and residues in the neighboring jR2R3-P301L chain (x-axis) for the structures selected in Region II from Figure 4 of the main text.

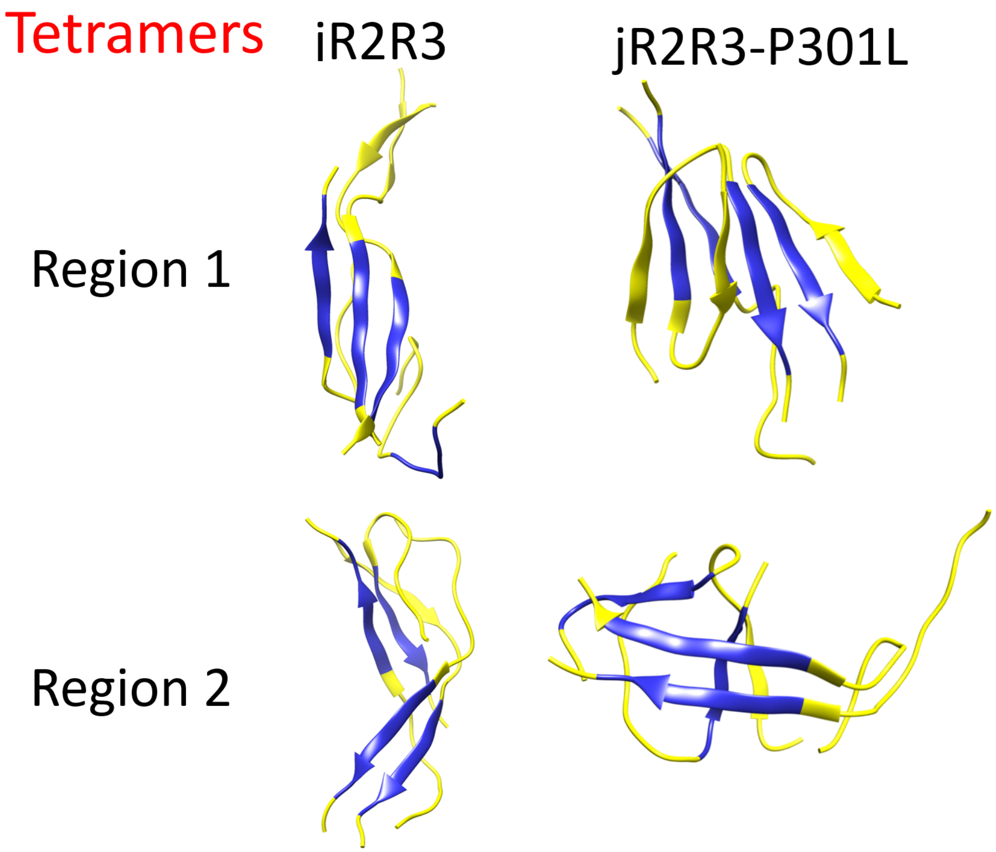

Figure S6: Representative full-complex conformations are shown for the jR2R3 and jR2R3-P301L tetramers extracted from the two most populated regions of the ELViM phase space. The PHF6 motif is highlighted in blue.
